# University Students’ Vaccination Intention after the Fifth Wave of the COVID-19 Outbreak in Hong Kong: Inspiration from a Health Belief Model

**DOI:** 10.3390/healthcare12121204

**Published:** 2024-06-15

**Authors:** Lu Hua Chen, Victoria Ka-Ying Hui, Yi-Ching Victoria Lai, Richard Huan Xu, Yingqi Guo

**Affiliations:** 1 Department of Rehabilitation Sciences, Faculty of Health and Social Sciences, The Hong Kong Polytechnic University, Hong Kong SAR, China; yi-ching-victoria.lai@polyu.edu.hk (Y.-C.V.L.); richard.xu@polyu.edu.hk (R.H.X.); 2 Research Institute for Smart Ageing (RISA), The Hong Kong Polytechnic University, Hong Kong SAR, China; 3 Mental Health Research Center (MHRC), The Hong Kong Polytechnic University, Hong Kong SAR, China; 4 Faculty of Social Sciences, The University of Hong Kong, Hong Kong SAR, China; vkyhui18@hku.hk; 5 Department of Social Work, Hong Kong Baptist University, Hong Kong SAR, China; yingqiguo@hkbu.edu.hk; 6 Department of Geography (Joint), Hong Kong Baptist University, Hong Kong SAR, China; 7 Smart Society Lab., Hong Kong Baptist University, Hong Kong SAR, China

**Keywords:** COVID-19 vaccination intention, Health Belief Model, influenza vaccine uptake, government vaccination lottery program, university students

## Abstract

The fifth wave of COVID-19, driven by the Omicron variant, started to surge in Hong Kong in December 2021. Previous studies have shown that younger adults, compared to older adults, are vulnerable to increased risks of side effects after vaccination. However, little is known about the COVID-19 vaccination behavior among younger adults, especially university students, in Hong Kong. Therefore, the present online survey study aimed to investigate the predictors of COVID-19 vaccination intention among university students in Hong Kong using the Health Belief Model (HBM) as a framework. Two other potential predictors, the previous influenza vaccine uptake frequency and the Hong Kong SAR government vaccination lottery program, were also examined. The intention to receive another dose of the COVID-19 vaccine was low (36.4%). Multivariate binomial logistic regression analysis showed that, after controlling for demographic and baseline characteristics, the perceived susceptibility (OR = 2.98, CI = 1.18–7.53) and previous influenza vaccine uptake frequency (OR = 1.54, CI = 1.08–2.19) significantly and positively predicted the COVID-19 vaccination intention. However, the government vaccination lottery program (i.e., wining prizes for being vaccinated) (OR = 0.87, CI = 0.34–2.26) was not a significant motivator of COVID-19 vaccination. Future public health campaigns should focus on the individual susceptibility to COVID-19 and past influenza vaccination history to promote increased vaccination uptake among university students.

## 1. Introduction

The outbreak of COVID-19 in 2019 led to an unprecedented public health crisis globally, characterized by substantial mortality rates [1]. In Hong Kong, the emergence of new COVID-19 variants, including Omicron, has been observed since the fifth wave of the pandemic in December 2021. The Omicron variant, characterized by extensive spike protein mutations, has a more expeditious infection rate than previous variants, such as Delta. Despite its seemingly lower mortality rate, Omicron could evade neutralizing antibodies from prior infection or vaccination, leading to rapid spread [2,3,4,5]. However, in Hong Kong, a significant portion of the population remains unvaccinated. Over 200 deaths per day were reported locally during the fifth wave of the pandemic, largely attributed to the lower full-vaccine coverage compared to the neighboring regions [6,7]. Of these deaths, 73% were among the unvaccinated, and only 6% had received a booster shot [8]. To mitigate the disease’s impact, the Hong Kong SAR government initiated a territory-wide vaccination scheme, offering two vaccines, “BioNTech” and “Sinovac Biotech” [9], to minimize disease complications.

However, vaccination intention is hindered by vaccine hesitancy, which refers to reluctance or a delay in accepting vaccination despite the availability of resources and services. Indeed, vaccine hesitancy has become a significant obstacle to achieving herd immunity, which requires at least 60% vaccine coverage [10]. Therefore, understanding the factors underlying hesitant behavior is crucial, as vaccination intentions are linked to a greater likelihood of subsequent vaccination behavior. In Hong Kong, because it remained challenging to encourage the vaccine-hesitant population to be vaccinated following the fifth wave of COVID-19, the Hong Kong SAR government adopted a mandatory policy known as the vaccine pass campaign (https://www.mayerbrown.com/en/insights/publications/2022/02/hong-kong-vaccine-pass-and-covid-related-restrictions-at-a-glance, accessed on 1 May 2022) to promote COVID-19 vaccination among the public. The mandatory vaccination policy, which contradicts individuals’ decision-making autonomy, has not only raised ethical arguments [11] but also impacted individuals’ vaccine hesitancy and subsequent vaccination behavior. However, so far, little research in Hong Kong has investigated this issue.

Age is a reported factor influencing vaccine hesitancy. Some studies suggest that young adults may be more vaccine-hesitant, although the specific dynamics within this age group are not fully understood [12]. The COVID-19 vaccines have higher efficacy in young populations, as demonstrated by the higher antibody levels post-vaccination; however, young adults are more susceptible to both local and systemic side effects than older adults [13]. This may be partially due to their enhanced ability to fight infections and their overactive responses to vaccines [14]. Furthermore, concerns over side effects [15] and debates around natural immunity versus vaccination [16] may have led some young adults to question the necessity, utility, and effectiveness of the boosters. Therefore, there is a need to examine the perceptions of the young population towards COVID-19 vaccines, especially boosters.

The Health Belief Model (HBM) is a widely recognized theory for the prediction of preventive health behavior [17,18]. The HBM regarding vaccination includes five key constructs: perceived severity (the consequences of infection), perceived susceptibility (the risk of infection), perceived benefits (the expected reduction in infection severity), perceived barriers (vaccination risks and potential psychosocial difficulties), and cues to action (information received from family and friends) [19,20]. Additionally, significant correlations have been reported between previous vaccination behavior, such as that regarding seasonal influenza and the 2009 H1N1 vaccine, and COVID-19 vaccine adherence in various populations, suggesting that individuals may form a habit of being vaccinated against diseases with similar modes of transmission [21,22,23]. However, little is known about past vaccination behavior and COVID-19 vaccine intention among university students in Hong Kong.

In Hong Kong, private companies initially introduced incentives, such as a lottery for a luxury apartment valued at USD 1.4 million, to enhance the COVID-19 vaccine coverage amidst the fifth wave of the pandemic [24]. Subsequently, the Hong Kong SAR government also began offering various incentives, including lotteries for prizes, exemptions from regular virus testing, vaccine passport policies for permission to enter bars and clubs, and reduced quarantine durations, to boost the COVID-19 vaccine coverage [25,26]. While similar initiatives, such as the Vax-a-million lottery event in Ohio, USA, have successfully increased the vaccination rates [27], the effectiveness of these lottery-based incentive strategies in promoting COVID-19 vaccine uptake among university students in Hong Kong remains uncertain and under-researched.

Thus, this study aimed to fill the aforementioned research gaps with two research objectives. The first objective was to investigate whether the key HBM constructs would predict university students’ intention to receive another dose of the COVID-19 vaccine after the fifth wave of the pandemic in Hong Kong. The second objective was to explore the potential roles of another two predictors, namely the previous influenza vaccine uptake frequency and the Hong Kong SAR government’s vaccination lottery program, in predicting university students’ vaccination intention.

## 2. Materials and Methods

### 2.1. Study Design and Data Source

This cross-sectional online survey study used convenience sampling to recruit participants from 28 May 2022 to 30 September 2022. The online survey was developed using the Qualtrics software (https://www.qualtrics.com/au/, accessed on 30 May 2022). The online survey was firstly pilot tested to evaluate the feasibility of the study. Based on the feedback from the participants of the pilot study, the questionnaire was further refined and improved. Subsequently, the online survey was disseminated through various social media platforms, including Facebook, Twitter, and Instagram (see Supplementary Materials for details). Sample size calculation was conducted using G*power 3.1. Assuming 80% power, a Type I error of 0.05, and a binomial distribution of 0.5, a minimum of 164 participants were required to detect an odds ratio of 2.5 with a 50% success rate.

This study originally recruited a total of 251 young adults aged between 18 and 26 who were pursuing a university degree or equivalent in Hong Kong. After excluding those with incomplete data (*n* = 85) and missing consent (*N* = 1), the final sample comprised 165 students from Hong Kong. This study received ethical approval from the Hong Kong Polytechnic University Institutional Review Board (Reference No.: HSEARS20220517005). Participation was entirely voluntary, with no incentives offered. Informed consent was obtained from all participants.

### 2.2. Measures

The online survey consisted of five sections. The first section collected information on participants’ demographic and background characteristics, such as age, gender, perceived general health, acceptance of vaccines in general (including influenza, Hepatitis B, and HPV vaccines), history of COVID-19 infection, total dosage of COVID-19 vaccine received, whether they lived with any vulnerable family members (e.g., the elderly or immunocompromised individuals), and whether they had family members, relatives, or close friends who had been affected by or died from COVID-19.

The second section pertained to the five constructs of the HBM, which is a reliable and valid measurement tool for the study of health-related behaviors [28,29] (see Supplementary Materials): (1) a 4-item measure of the perceived severity and consequences of contracting COVID-19 (concerns over the impact and severity of COVID-19); (2) a 7-item measure of the perceived susceptibility to COVID-19 (likelihood of contracting the virus); (3) a 5-item measure of the perceived benefits of receiving the COVID-19 vaccine (likelihood of protection from the vaccine); (4) an 8-item measure of the perceived barriers to and concerns about receiving the COVID-19 vaccine (concerns over vaccine side effects and doubts about vaccine safety and efficacy); and (5) a 6-item measure of cues to action (information provided by different parties that inform vaccine decision-making, such as recommendations by health authorities, family, or peers). Each construct was rated on a 5-point Likert scale, ranging from 1 (*strongly disagree*) to 5 (*strongly agree*).

The third section assessed participants’ previous influenza vaccination behavior by asking them to indicate the frequency of their influenza vaccine uptake (*seldom or never, not regular, almost every year, or every year*). The fourth section inquired about whether the Hong Kong SAR government’s vaccination lottery program had increased their motivation to receive the COVID-19 vaccine (*yes/no*). The fifth section asked participants about their intention to receive another dose of the COVID-19 vaccine following the 5th wave of COVID-19 (*yes/no*).

### 2.3. Data Analysis

Statistical analysis was performed using Jamovi (version 2.2.5) (https://www.jamovi.org/, accessed on 30 May 2022) by the second author, who was blinded to the study protocol and was not involved in the data collection. Descriptive statistics were computed for the demographic and background characteristics, as well as for the variables of interest. The primary outcome variable was a dichotomous variable representing participants’ intention to receive another dose of the COVID-19 vaccine. The univariate binomial logistic regression results were based on Pearson’s chi-square tests. Multivariate binomial logistic regression was conducted with vaccination intention as the outcome variable, while controlling for participants’ demographic and background characteristics, with *p* values < 0.05. Four hierarchical multivariate binomial logistic regression models were obtained with (1) demographic and baseline characteristics; (2) demographic and baseline characteristics as well as HBM constructs; (3) demographic and baseline characteristics as well as the frequency of previous influenza vaccine uptake; and (4) demographic and baseline characteristics as well as the perceived increased incentive to receive a COVID-19 vaccine from the Hong Kong SAR government’s vaccination lottery program. A *p* value < 0.05 was regarded as statistically significant.

## 3. Results

### 3.1. Participants’ Characteristics

The final sample consisted of 64 male (38.79%) and 101 female (61.21%) students (see Table 1). The majority were aged between 18 and 21 (83.64%), perceived themselves as healthy (53.33%), and had not contracted COVID-19 (65.45%). Although most of them received three doses of the COVID-19 vaccine (73.33%) and were accepting of vaccines in general (such as influenza vaccines, Hepatitis B vaccines, and HPV vaccines) (55.76%), they seldom or never received influenza vaccines (67.27%). The majority were not living with vulnerable family members who were elderly or had weak immune systems (80.61%), were not close to anyone who had contracted COVID-19 (86.67%), and were not close to anyone who had died from COVID-19 (96.36%). 

### 3.2. Intention to Receive Another Dose of COVID-19 Vaccine

Overall, 36.37% of the participants intended to receive another dose of the COVID-19 vaccine. The descriptive statistics for the variables of interest are displayed in Table 2.

The univariate binomial logistic regression showed that participants who had more frequent influenza vaccine uptake (*OR* = 1.52, *p* = 0.01), who perceived the consequences of having COVID-19 to be more severe (*OR* = 1.93, *p* = 0.02), who perceived themselves to have a higher susceptibility to COVID-19 (*OR* = 2.28, *p* = 0.03), who perceived more benefits of receiving a COVID-19 vaccine (*OR* = 2.08, *p* < 0.001), and who perceived fewer barriers to receiving a COVID-19 vaccine (*OR* = 0.50, *p* = 0.005) had a higher intention to receive another dose of the COVID-19 vaccine (see Table 3).

The multivariate hierarchical binomial logistic regression showed that, after controlling for demographic and background characteristics, participants who perceived themselves to have a higher susceptibility to COVID-19 (*OR* = 2.98, *p* = 0.02) and had more frequent influenza vaccine uptake (*OR* = 1.54, *p* = 0.02) had a significantly higher intention to receive another dose of the COVID-19 vaccine (see Table 4). In contrast, after controlling for demographic and background characteristics, the perceived increased incentive from the Hong Kong SAR government’s vaccination lottery program was not a significant predictor of participants’ intention to receive another dose of the COVID-19 vaccine (*OR* = 0.87, *p* = 0.78) (see Table 4).

## 4. Discussion

In contrast to the 60% intention rate among American and Vietnamese students [30], Hong Kong university students’ intention to receive another dose of the COVID-19 vaccine was rather low (36.4%) in the present study. However, such findings are consistent with previous studies showing that individuals aged 18–24 were the most unwilling group to receive the COVID-19 vaccine [31,32]. In Hong Kong, COVID-19 vaccination is mandatory to enter different public venues—for example, schools and restaurants—which may explain the high vaccination rate (99.39%) and low vaccination intention (36.37%) identified in the present study. 

Using univariate logistic regression, four HBM constructs (perceived susceptibility, perceived severity, perceived benefits, and perceived barriers) significantly predicted COVID-19 vaccination intention. These findings were consistent with those of previous studies on Chinese and other Asian populations during the same period [33,34,35], where the perceived severity and perceived benefits were positively associated with vaccination intention and perceived barriers were negatively associated with it. Likewise, high perceived susceptibility, high perceived benefits, and low perceived barriers were reported to be significant predictors of COVID-19 vaccination intention in previous studies [36,37]. It is speculated that university students consider COVID-19 vaccines as a means to help society to return to normal by reducing the severity of infection and preventing the spread of COVID-19 [38,39]. Moreover, the present findings provide additional evidence supporting previous research about Hong Kong university students’ preference for vaccines, in which they were most concerned about the efficacy of the vaccine against COVID-19 infection but were generally willing to take a certain level of risk as a trade-off for better protection [40].

Perceived susceptibility remained significant in predicting COVID-19 vaccination intention in the multivariate hierarchical logistic regression model (Model 2). The positive association between perceived susceptibility and vaccination intention is in line with previous findings indicating that individuals who perceived a higher risk of contracting COVID-19 were more likely to receive a vaccine [36,38,41]. During the fifth wave of the COVID-19 outbreak in Hong Kong, a large number of infections were estimated to be unreported due to the surge in asymptomatic and mild cases [42]. Therefore, worrying about the high risk of infection by Omicron (perceived susceptibility) served as a significant motivator of university students’ vaccination behavior, as highlighted by the present findings. The significant positive association between perceived susceptibility and vaccination intention in the present study underscores the importance of perceived risks in health behavior theory, which posits that individuals who perceive a higher likelihood of contracting an illness are more inclined to engage in behavior to prevent it [43]. In contrast, previous findings from Lin et al. and Wong et al. [17,44] showed that perceived susceptibility was not a significant predictor of COVID-19 vaccination intention. This might be because the timeframes of the previous surveys were situated around 2020 to 2021 (the initial stage of the COVID-19 pandemic), differing from the timeframe of the present study (after the fifth wave of the pandemic in 2022). Moreover, the present study focused on a particular age group (young adults aged between 18 and 26), which may also have contributed to these discrepancies. 

Among the five HBM constructs, cues to action were the only nonsignificant predictor. By the time of the fifth wave of the COVID-19 outbreak in Hong Kong, it is believed that university students had already formed pre-existing beliefs about the COVID-19 vaccine based on their previous experiences. When vaccination became more common and was later made mandatory by the government, the impact of additional cues to encourage vaccination might have diminished. Moreover, vaccination fatigue, which refers to inaction towards vaccine instruction or information, could also be a reason that the vaccinated young population were less responsive to cues. As a result of the perceived burnout or burden of being frequently immunized, individuals might start to intentionally avoid news related to health preventive measures [45,46]. Thus, cues to action did not significantly predict university students’ intention to receive another dose of the COVID-19 vaccine, as demonstrated by the present study. 

Apart from the HBM constructs, the frequency of previous influenza uptake was found to be a significant predictor of COVID-19 vaccination intention in the multivariate hierarchical logistic regression model (Model 3). The studies conducted by Shmueli et al. and Le et al. [30,47] demonstrated that individuals who had received frequent seasonal influenza vaccines had higher COVID-19 vaccination intention, which resonates with the present findings. It is likely that individuals who receive influenza vaccines frequently are more concerned and cautious about infectious diseases, or they are more confident about the safety of the vaccines. Ohtomo et al. [48] explained that past preventive behavior and related habits could trigger the automated execution of future preventive behavior, which, in turn, positively influences behavioral intention. It is also likely that young adults consider past experiences of seasonal influenza vaccination as effective protection against the adverse consequences of contracting influenza, which may help to reduce the severity of respiratory diseases including COVID-19. 

This is the first study to investigate the effects of the vaccination lottery program provided by the Hong Kong SAR government on university students’ intention to receive another dose of the COVID-19 vaccine. In contrast to earlier findings that demonstrated that incentives could have a considerable impact in terms of boosting the vaccination rates, especially on those who were younger and doubted the necessity of vaccination [49], the vaccination lottery program implemented by the Hong Kong SAR government was ineffective in boosting vaccination intention among university students, as only 15.76% of them perceived an increased incentive from the program. While guaranteed cash payments have been shown to successfully increase the COVID-19 vaccination rate in countries like Sweden [50], they have not been offered in Hong Kong [25]. Because only a few individuals can win the prize from the government’s vaccination lottery program, such lottery-based incentives have become less appealing to young adults, including university students, as suggested by the present study. 

Our study findings have important implications for public health campaigns against COVID-19. Given that four HBM constructs significantly predicted COVID-19 vaccination intention, it is recommended that public health communication efforts should emphasize these constructs to promote vaccination behavior among university students. At present, the free-of-charge influenza vaccine covers only up to 18-year-old secondary school students, which is insufficient to motivate young adults to receive the influenza vaccine in Hong Kong [51]. The provision of more funding is, thus, recommended to improve the COVID-19 vaccination rate for those university students who do not frequently receive the influenza vaccine. This is because the present study found that the more frequently the university students received the seasonal influenza vaccine, the more likely they were to receive another dose of the COVID-19 vaccine. Moreover, as quarantine-free travel was reported to be strongly preferred by university students in Hong Kong [40], such immediate and guaranteed incentive programs that cater to the needs of young adults could be established to increase university students’ COVID-19 vaccination uptake. 

There are several limitations of the present study. First, due to the rapid changes in quarantine and social distancing policies and measures, this cross-sectional online survey study might not have been able to capture the changes in COVID-19 vaccination intention across time, or to demonstrate a causal relationship between the variables. In future studies, it is recommended to adopt a longitudinal design to conduct a trend analysis of the COVID-19 vaccination intention. Second, this study relied on participants’ self-reports. Since intention does not necessarily translate into action, future studies are recommended that combine other objective measures to investigate actual vaccination behavior—for example, by observing whether participants have taken action towards vaccine reservation [50]. Third, different types of vaccines, which vary in their degree of protection, safety profile, and side effects [52], may influence individuals’ intentions regarding COVID-19 vaccination. In Hong Kong, two types of vaccines, “BioNTech“ and ”Sinovac Biotech“, were offered after the fifth wave of the COVID-19 outbreak. However, due to the limited sample size, we did not investigate the association between the vaccine type and vaccination intention. A future study should explore the specific vaccines’ impacts based on a larger sample size. Lastly, selection bias might have arisen due to the use of convenience sampling in our study design. This sampling technique, while effective for initial insights, might not fully represent the diverse opinions and behavior of all university students in Hong Kong. Future studies should employ a random sampling method to achieve a more representative population distribution.

## 5. Conclusions

In summary, the present study suggests that HBM constructs in the domains of perceived severity, susceptibility, benefits, and barriers are significant predictors of university students’ COVID-19 vaccination intention. Moreover, the previous influenza vaccine uptake frequency, but not the Hong Kong SAR government’s vaccination lottery program, significantly predicted higher COVID-19 vaccination intention. Future public health campaigns should focus on the individual susceptibility to COVID-19 and past influenza vaccination history to promote increased vaccination uptake among university students.

## Figures and Tables

**Table 1:** Demographic and background characteristics of participants (*N* = 165).

Characteristic	Category	n	(%)
Age ^a^ (years old)	18–21	138	(83.64%)
	22–26	25	(15.15%)
	Missing	2	(1.21%)
Gender	Male	64	(38.79%)
	Female	101	(61.21%)
Living with vulnerable family members	No	133	(80.61%)
	Yes	32	(19.39%)
Perceived general health	Very unhealthy	0	(0.00%)
	Unhealthy	11	(6.67%)
	Fair	50	(30.30%)
	Healthy	88	(53.33%)
	Very healthy	16	(9.70%)
History of COVID-19	No	108	(65.45%)
	Yes	57	(34.55%)
Being close to someone who had contracted COVID-19	No	22	(86.67%)
	Yes	143	(13.33%)
Being close to someone who had died from COVID-19	No	159	(96.36%)
	Yes	6	(3.64%)
COVID-19 vaccine dosage	0 dose	1	(0.61%)
	1 dose	2	(1.21%)
	2 doses	37	(22.42%)
	3 doses	121	(73.33%)
	4 doses	4	(2.42%)
Frequency of influenza vaccine uptake	Seldom or never	111	(67.27%)
	Not regular	24	(14.55%)
	Almost every year	15	(9.09%)
	Every year	15	(9.09%)
Acceptance of vaccines in general (such as influenza vaccines, Hepatitis B vaccines, and HPV vaccines)	No	73	(44.24%)
	Yes	92	(55.76%)

Note. ^a^ The mean age was 20.3 years old (*SD* = 1.5), ranging from 18 to 26.

**Table 2:** Descriptive statistics for variables of interest (*N* = 165).

Characteristic	Category	Mean	*SD*	Cronbach’s Alpha
HBM constructs				
Perceived severity	-	3.80	0.64	0.70
Perceived susceptibility	-	3.32	0.46	0.71
Perceived benefits	-	2.83	0.88	0.88
Perceived barriers	-	3.69	0.70	0.87
Cues to action	-	2.67	0.63	0.80
Frequency of influenza vaccine uptake	-	1.60	0.99	-
		***n***	**(%)**	
Perceived increased incentive from government’s vaccination lottery program	No	139	(82.24%)	-
	Yes	26	(15.76%)	-
Intention to receive another dose of COVID-19 vaccine	No	105	(63.63%)	-
	Yes	60	(36.37%)	-

Note. HBM = Health Belief Model; *SD* = standard deviation.

**Table 3:** Univariate binomial logistic regression predicting intention to receive another dose of COVID-19 vaccine ^a^ (*N* = 165).

Characteristic	Category	*OR*	95% CI	*p*
Age	-	1.10	0.89–1.37	0.37
Perceived general health	-	1.12	0.73–1.72	0.61
COVID-19 dosage	-	1.40	0.75–2.60	0.29
Gender (reference = male)	Female	0.92	0.48–1.77	0.81
Living with vulnerable family members (reference = no)	Yes	1.47	0.67–3.22	0.34
History of COVID-19 (reference = no)	Yes	0.92	0.47–1.80	0.81
Being close to someone who had contracted COVID-19 (reference = no)	Yes	1.00	0.39–2.54	1.00
Being close to someone who had died from COVID-19 (reference = no)	Yes	0.34	0.04–2.97	0.33
Acceptance of vaccines in general (such as influenza vaccines, Hepatitis B vaccines, and Human Papillomavirus vaccines) (reference = no)	Yes	1.06	0.56–2.01	0.86
Perceived severity	-	**1.93**	**1.12–3.33**	**0.02**
Perceived susceptibility	-	**2.28**	**1.09–4.74**	**0.03**
Perceived benefits	-	**2.08**	**1.37–3.15**	**<0.001**
Perceived barriers	-	**0.50**	**0.31–0.81**	**0.005**
Cues to action	-	1.03	0.62–1.70	0.91
Frequency of previous influenza vaccine uptake	-	**1.52**	**1.10–2.10**	**0.01**
Perceived increased incentive from government’s vaccination lottery program (reference = no)	Yes	1.11	0.47–2.64	0.81

Note. ^a^ Reference = no intention to receive another dose of COVID-19 vaccine. OR = odd ratio; CI = confidential interval.

**Table 4:** Multivariate binomial logistic regression predicting intention to receive another dose of COVID-19 vaccine ^a^ (*N* = 165).

		Model 1			Model 2			Model 3			Model 4		
Characteristic	Category	*OR*	95% CI	*p*	*OR*	95% CI	*p*	*OR*	95% CI	*p*	*OR*	95% CI	*p*
Age	-	1.11	0.89–1.38	0.34	1.06	0.83–1.37	0.63	1.11	0.89–1.39	0.34	1.11	0.90–1.39	0.33
Perceived general health	-	1.24	0.78–1.97	0.36	1.45	0.86–2.47	0.17	1.20	0.75–1.92	0.46	1.23	0.78–1.96	0.37
COVID-19 dosage	-	1.32	0.63–2.77	0.47	1.07	0.47–2.46	0.87	1.30	0.61–2.78	0.49			
Gender (reference = male)	Female	1.07	0.51–2.26	0.86	0.99	0.42–2.30	0.98	1.13	0.53–2.42	0.75	1.06	0.50–2.24	0.89
Living with vulnerable family members (reference = no)	Yes	1.18	0.51–2.71	0.70	1.23	0.48–3.16	0.66	1.14	0.49–2.70	0.76	1.21	0.51–2.83	0.67
History of COVID-19 (reference = no)	Yes	1.07	0.50–2.29	0.86	0.85	0.36–2.02	0.72	0.95	0.43–2.09	0.90	1.08	0.50–2.31	0.85
Being close to someone who had contracted COVID-19 (reference = no)	Yes	0.88	0.33–2.35	0.79	0.90	0.30–2.73	0.85	1.12	0.40–3.16	0.83	0.88	0.33–2.38	0.81
Being close to someone who had died from COVID-19 (reference = no)	Yes	0.34	0.04–3.35	0.36	0.16	0.01–2.21	0.17	0.41	0.04–4.08	0.45	0.34	0.03–3.31	0.35
Acceptance of vaccines in general (such as influenza vaccines, Hepatitis B vaccines, and HPV vaccines) (reference = no)	Yes	1.05	0.51–2.19	0.89	0.92	0.42–2.03	0.84	0.79	0.36–1.72	0.55	1.05	0.51–2.19	0.89
Perceived severity		-	-	-	1.98	1.00–3.94	0.051	-	-	-	-	-	-
Perceived susceptibility		-	-	-	**2.98**	**1.18–7.53**	**0.02**	-	-	-	-	-	-
Perceived benefits		-	-	-	1.58	0.87–2.87	0.14	-	-	-	-	-	-
Perceived barriers		-	-	-	0.58	0.29–1.16	0.13	-	-	-	-	-	-
Cues to action		-	-	-	0.53	0.27–1.06	0.07	-	-	-	-	-	-
Influenza vaccine uptake		-	-	-	-	-	-	**1.54**	**1.08–2.19**	**0.02**	-	-	-
Perceived increased incentive from government’s vaccination lottery program (reference = no)	Yes	-	-	-	-	-	-	-	-	-	0.87	0.34–2.26	0.78
*χ* ^2^		3.70			**30.73**			9.51			3.78		
Df		9			**14**			10			10		
*p*		0.93			**0.006**			0.48			0.96		
Δ*χ*^2^		-			**27.03**			**5.82**			0.08		
Δdf		-			**5**			**1**			1		
*p*		-			**<0.001**			**0.02**			0.78		

Note. ^a^ Reference = no intention to receive another dose of COVID-19 vaccine. Model (1) variables: age, perceived general health, COVID-19 dosage, gender, living with vulnerable family members, history of COVID-19, being close to someone who had contracted COVID-19, being close to someone who had died from COVID-19, acceptance of vaccines in general (such as influenza vaccines, Hepatitis B vaccines, and Human Papillomavirus vaccines). Model (2) variables: Model (1) variables and Health Belief Model constructs. Model (3) variables: Model (1) variables and frequency of previous influenza vaccine uptake. Model (4) variables: Model (1) variables and perceived increased incentive from the government’s vaccination lottery program. OR = odds ratio; CI = confidence interval.

## Data Availability

All data are available from the corresponding author upon request.

## Supplementary Materials

The following supporting information can be downloaded at: https://www.mdpi.com/article/10.3390/healthcare12121204/s1.
