# Supplementary material for: University Students’ Vaccination Intention after the Fifth Wave of the COVID-19 Outbreak in Hong Kong: Inspiration from a Health Belief Model
Source: Healthcare (Basel). 2024 Jun 15;12(12):1204. doi: 10.3390/healthcare12121204 (PMC11203942; doi:10.3390/healthcare12121204)
Supplement: Supplementary file 1 [file healthcare-12-01204-s001.zip › healthcare-3016141-supplementary.pdf]

Supplementary Materials

Survey development and dissemination procedure

The online survey was developed using Qualtrics software. The online survey was firstly pilot tested to evaluate the feasibility of the study. Based on the feedback from participants of the pilot study, the questionnaire was further refined and improved. Subsequently, the online survey was disseminated through various social media platforms, including Facebook, Twitter, and Instagram.

Content of online survey

Introduction

The research aims to study the intention of receiving COVID-19 vaccination of students through conducting an online survey. The findings could provide insights on public health education and promotion strategies for preventing future infectious diseases in Hong Kong. The questionnaire will not ask about your privacy. Participating in this research will not cause any risks, harm, or inconvenience. The survey will take approximately 20-25 minutes to complete.

Health Belief Model Constructs

1 = Strongly disagree
2 = Disagree
3 = Neutral
4 = Agree
5 = Strongly agree

Perceived severity

1. If I was infected with COVID-19, it may affect my study performance.
2. For the majority, COVID-19 leads to mild illnesses, but it can also cause severe illnesses for the others.
3. Some variants of COVID-19 e.g. Delta variant can be fatal.
4. I believe COVID-19 is more severe than seasonal influenza.

Perceived susceptibility to COVID-19 infection

1. I am at risk of being infected by COVID-19.
2. My household members are at risk of being infected by COVID-19.
3. I feel knowledgeable about my risks of being infected by COVID-19.
4. I am susceptible to being infected due to my exposure.
5. Healthy people can get COVID-19.
6. It is easier for me to get COVID-19 due to my health status.
7. I think I can be more able to protect myself against COVID-19 compared to other people.

Perceived benefits of receiving COVID-19 vaccines

1. The COVID-19 vaccines will help return to normal life.
2. COVID-19 vaccines will reduce the chance of getting COVID-19.
3. Vaccination is a good idea because it makes me feel less concerned about getting COVID-19.
4. COVID-19 vaccines can reduce the severity of COVID-19.
5. When I get COVID-19 vaccines, the whole society benefits from preventing the spread of COVID-19.

Perceived barriers to receiving COVID-19 vaccines

1. I may die after getting COVID-19 vaccines.

2. I am worried about the novelty of COVID-19 vaccines.
3. I am worried about the efficacy of COVID-19 vaccines.
4. I am worried about the safety of COVID-19 vaccines (e.g. contamination).
5. I am worried about the reliability of the manufacturer and the source of supply.
6. I am worried about the vaccine frequency (number of doses) required.
7. I am worried about the duration of immunity (to what extent I will be protected).
8. I am concerned about the possible side effects of COVID-19 vaccines, which will interfere with my daily activities.

Cues to action

1. I will get the COVID-19 vaccines if I was given adequate and reliable information about it.
2. I will get the COVID-19 vaccines if the vaccines are recommended by family members (i.e. parents, relatives, etc.).
3. I will get the COVID-19 vaccines if the vaccines are recommended by peers.
4. I will get the COVID-19 vaccines if the vaccines are recommended by the health authorities.
5. I will get COVID-19 vaccines if the vaccines are recommended by the media.
6. I will get the COVID-19 vaccines if the vaccines are taken by the majority.
